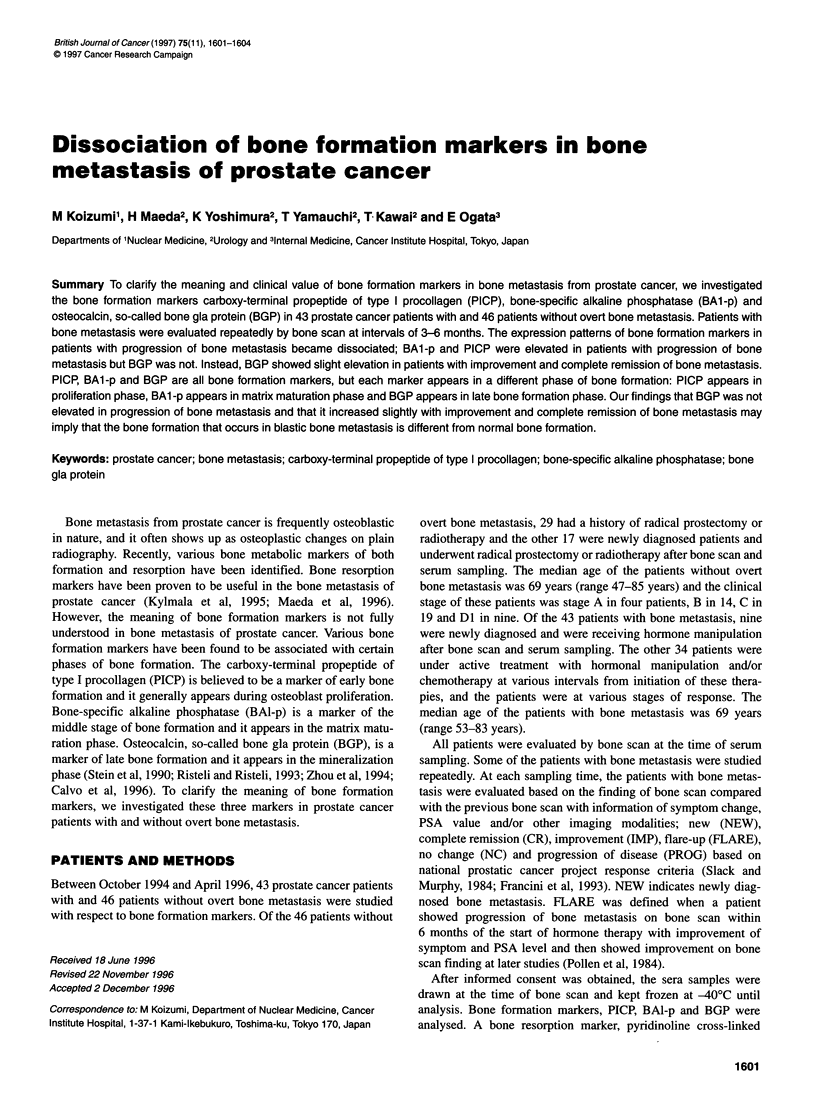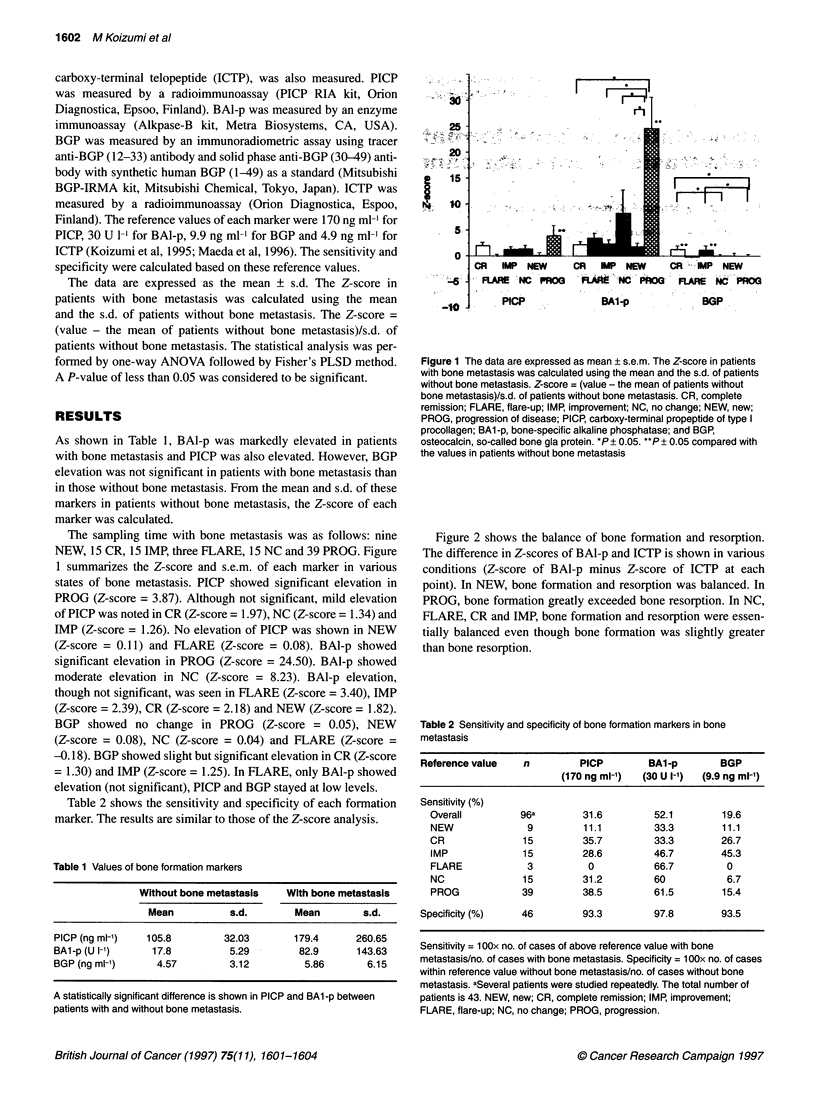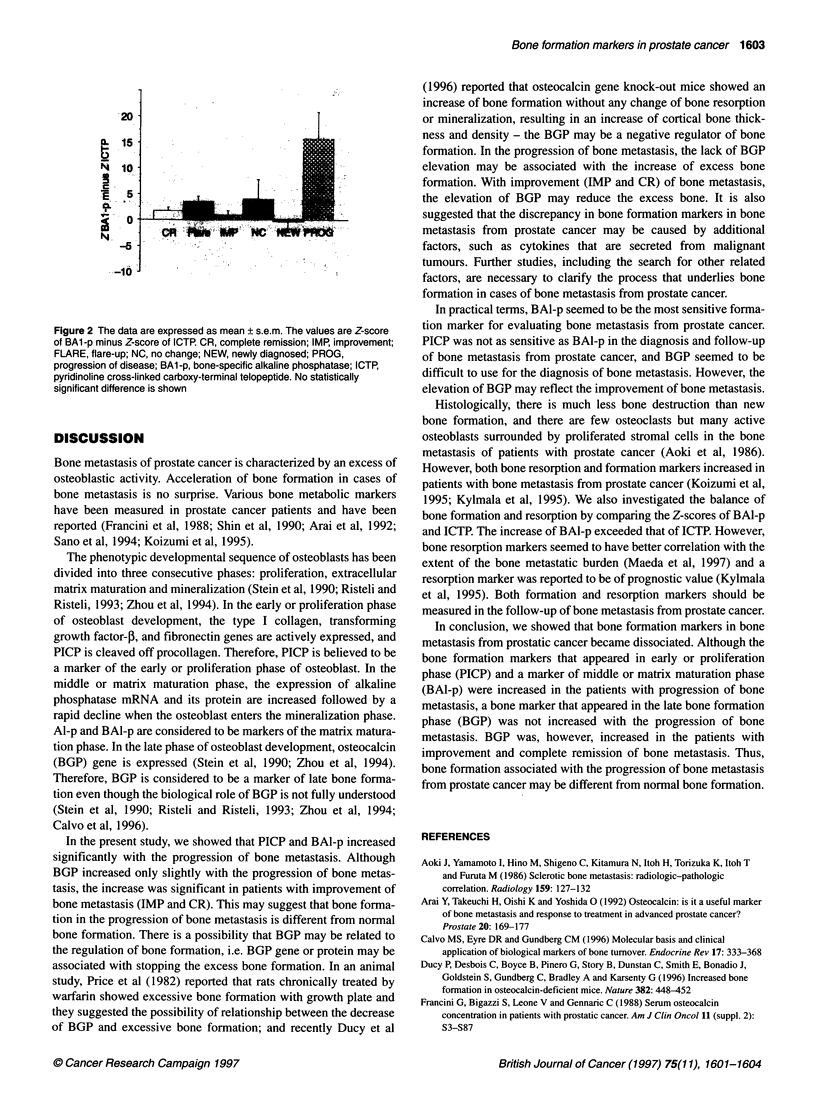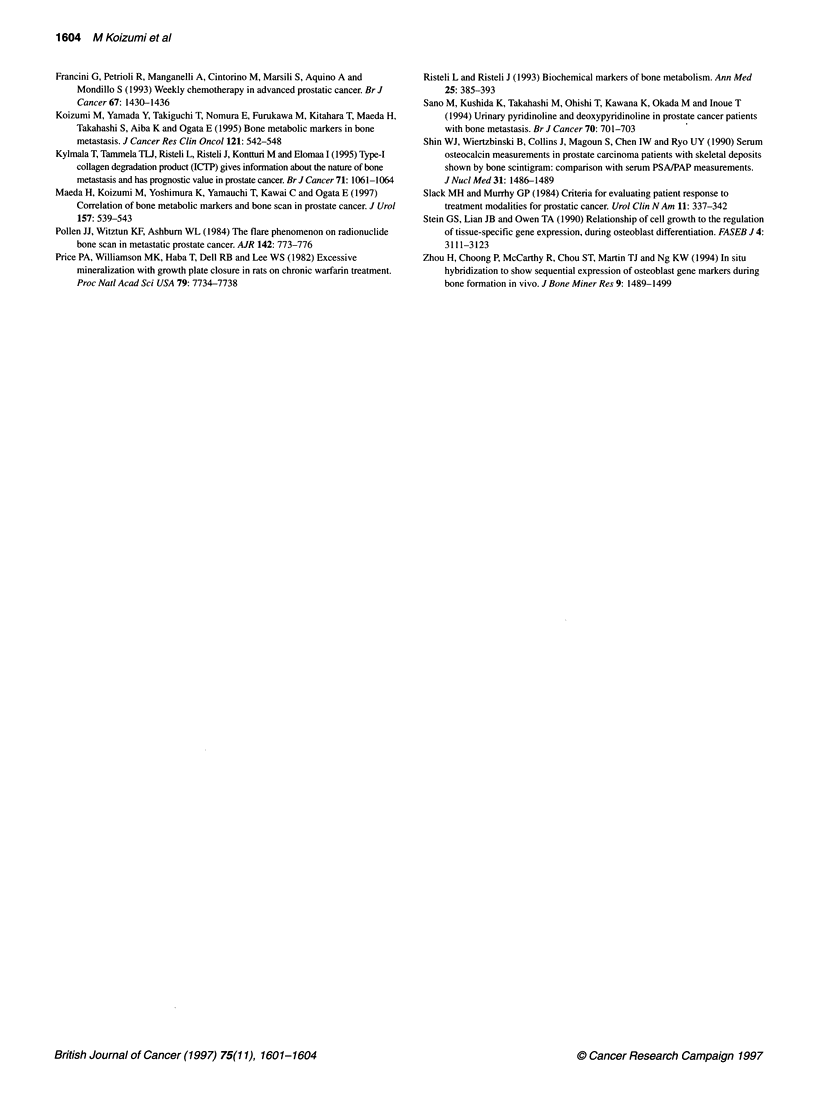# Dissociation of bone formation markers in bone metastasis of prostate cancer.

**DOI:** 10.1038/bjc.1997.273

**Published:** 1997

**Authors:** M. Koizumi, H. Maeda, K. Yoshimura, T. Yamauchi, T. Kawai, E. Ogata

**Affiliations:** Department of Nuclear Medicine, Cancer Institute Hospital, Toshima-ku, Tokyo, Japan.

## Abstract

To clarify the meaning and clinical value of bone formation markers in bone metastasis from prostate cancer, we investigated the bone formation markers carboxy-terminal propeptide of type I procollagen (PICP), bone-specific alkaline phosphatase (BA1-p) and osteocalcin, so-called bone gla protein (BGP) in 43 prostate cancer patients with and 46 patients without overt bone metastasis. Patients with bone metastasis were evaluated repeatedly by bone scan at intervals of 3-6 months. The expression patterns of bone formation markers in patients with progression of bone metastasis became dissociated; BA1-p and PICP were elevated in patients with progression of bone metastasis but BGP was not. Instead, BGP showed slight elevation in patients with improvement and complete remission of bone metastasis. PICP, BA1-p and BGP are all bone formation markers, but each marker appears in a different phase of bone formation: PICP appears in proliferation phase, BA1-p appears in matrix maturation phase and BGP appears in late bone formation phase. Our findings that BGP was not elevated in progression of bone metastasis and that it increased slightly with improvement and complete remission of bone metastasis may imply that the bone formation that occurs in blastic bone metastasis is different from normal bone formation.


British Journal of Cancer (1997) 75(11), 1601–1604
© 1997 Cancer Research Campaign

### Dissociation of bone formation markers in bone metastasis of prostate cancer

**M Koizumi[1], H Maeda[2], K Yoshimura[2], T Yamauchi[2], T Kawai[2] and E Ogata[3]**

Departments of [1]Nuclear Medicine, [2]Urology and [3]Internal Medicine, Cancer Institute Hospital, Tokyo, Japan

**Summary** To clarify the meaning and clinical value of bone formation markers in bone metastasis from prostate cancer, we investigated the bone formation markers carboxy-terminal propeptide of type I procollagen (PICP), bone-specific alkaline phosphatase (BA1-p) and osteocalcin, so-called bone gla protein (BGP) in 43 prostate cancer patients with and 46 patients without overt bone metastasis. Patients with bone metastasis were evaluated repeatedly by bone scan at intervals of 3–6 months. The expression patterns of bone formation markers in patients with progression of bone metastasis became dissociated; BA1-p and PICP were elevated in patients with progression of bone metastasis but BGP was not. Instead, BGP showed slight elevation in patients with improvement and complete remission of bone metastasis. PICP, BA1-p and BGP are all bone formation markers, but each marker appears in a different phase of bone formation: PICP appears in proliferation phase, BA1-p appears in matrix maturation phase and BGP appears in late bone formation phase. Our findings that BGP was not elevated in progression of bone metastasis and that it increased slightly with improvement and complete remission of bone metastasis may imply that the bone formation that occurs in blastic bone metastasis is different from normal bone formation.

**Keywords:** prostate cancer; bone metastasis; carboxy-terminal propeptide of type I procollagen; bone-specific alkaline phosphatase; bone gla protein

Bone metastasis from prostate cancer is frequently osteoblastic in nature, and it often shows up as osteoplastic changes on plain radiography. Recently, various bone metabolic markers of both formation and resorption have been identified. Bone resorption markers have been proven to be useful in the bone metastasis of prostate cancer (Kylmala et al, 1995; Maeda et al, 1996). However, the meaning of bone formation markers is not fully understood in bone metastasis of prostate cancer. Various bone formation markers have been found to be associated with certain phases of bone formation. The carboxy-terminal propeptide of type I procollagen (PICP) is believed to be a marker of early bone formation and it generally appears during osteoblast proliferation. Bone-specific alkaline phosphatase (BAl-p) is a marker of the middle stage of bone formation and it appears in the matrix maturation phase. Osteocalcin, so-called bone gla protein (BGP), is a marker of late bone formation and it appears in the mineralization phase (Stein et al, 1990; Risteli and Risteli, 1993; Zhou et al, 1994; Calvo et al, 1996). To clarify the meaning of bone formation markers, we investigated these three markers in prostate cancer patients with and without overt bone metastasis.

### PATIENTS AND METHODS

Between October 1994 and April 1996, 43 prostate cancer patients with and 46 patients without overt bone metastasis were studied with respect to bone formation markers. Of the 46 patients without overt bone metastasis, 29 had a history of radical prostectomy or radiotherapy and the other 17 were newly diagnosed patients and underwent radical prostectomy or radiotherapy after bone scan and serum sampling. The median age of the patients without overt bone metastasis was 69 years (range 47–85 years) and the clinical stage of these patients was stage A in four patients, B in 14, C in 19 and D1 in nine. Of the 43 patients with bone metastasis, nine were newly diagnosed and were receiving hormone manipulation after bone scan and serum sampling. The other 34 patients were under active treatment with hormonal manipulation and/or chemotherapy at various intervals from initiation of these therapies, and the patients were at various stages of response. The median age of the patients with bone metastasis was 69 years (range 53–83 years).

All patients were evaluated by bone scan at the time of serum sampling. Some of the patients with bone metastasis were studied repeatedly. At each sampling time, the patients with bone metastasis were evaluated based on the finding of bone scan compared with the previous bone scan with information of symptom change, PSA value and/or other imaging modalities; new (NEW), complete remission (CR), improvement (IMP), flare-up (FLARE), no change (NC) and progression of disease (PROG) based on national prostatic cancer project response criteria (Slack and Murphy, 1984; Francini et al, 1993). NEW indicates newly diagnosed bone metastasis. FLARE was defined when a patient showed progression of bone metastasis on bone scan within 6 months of the start of hormone therapy with improvement of symptom and PSA level and then showed improvement on bone scan finding at later studies (Pollen et al, 1984).

After informed consent was obtained, the sera samples were drawn at the time of bone scan and kept frozen at –40°C until analysis. Bone formation markers, PICP, BAl-p and BGP were analysed. A bone resorption marker, pyridinoline cross-linked

*Received 18 June 1996*
*Revised 22 November 1996*
*Accepted 2 December 1996*

*Correspondence to:* M Koizumi, Department of Nuclear Medicine, Cancer Institute Hospital, 1-37-1 Kami-Ikebukuro, Toshima-ku, Tokyo 170, Japan carboxy-terminal telopeptide (ICTP), was also measured. PICP was measured by a radioimmunoassay (PICP RIA kit, Orion Diagnostica, Epsoo, Finland). BAl-p was measured by an enzyme immunoassay (Alkpase-B kit, Metra Biosystems, CA, USA). BGP was measured by an immunoradiometric assay using tracer anti-BGP (12–33) antibody and solid phase anti-BGP (30–49) antibody with synthetic human BGP (1–49) as a standard (Mitsubishi BGP-IRMA kit, Mitsubishi Chemical, Tokyo, Japan). ICTP was measured by a radioimmunoassay (Orion Diagnostica, Espoo, Finland). The reference values of each marker were 170 ng $ml^{-1}$ for PICP, 30 U $l^{-1}$ for BAl-p, 9.9 ng $ml^{-1}$ for BGP and 4.9 ng $ml^{-1}$ for ICTP (Koizumi et al, 1995; Maeda et al, 1996). The sensitivity and specificity were calculated based on these reference values.

The data are expressed as the mean ± s.d. The *Z*-score in patients with bone metastasis was calculated using the mean and the s.d. of patients without bone metastasis. The *Z*-score = (value – the mean of patients without bone metastasis)/s.d. of patients without bone metastasis. The statistical analysis was performed by one-way ANOVA followed by Fisher's PLSD method. A *P*-value of less than 0.05 was considered to be significant.

### RESULTS

As shown in Table 1, BAl-p was markedly elevated in patients with bone metastasis and PICP was also elevated. However, BGP elevation was not significant in patients with bone metastasis than in those without bone metastasis. From the mean and s.d. of these markers in patients without bone metastasis, the *Z*-score of each marker was calculated.

The sampling time with bone metastasis was as follows: nine NEW, 15 CR, 15 IMP, three FLARE, 15 NC and 39 PROG. Figure 1 summarizes the *Z*-score and s.e.m. of each marker in various states of bone metastasis. PICP showed significant elevation in PROG (*Z*-score = 3.87). Although not significant, mild elevation of PICP was noted in CR (*Z*-score = 1.97), NC (*Z*-score = 1.34) and IMP (*Z*-score = 1.26). No elevation of PICP was shown in NEW (*Z*-score = 0.11) and FLARE (*Z*-score = 0.08). BAl-p showed significant elevation in PROG (*Z*-score = 24.50). BAl-p showed moderate elevation in NC (*Z*-score = 8.23). BAl-p elevation, though not significant, was seen in FLARE (*Z*-score = 3.40), IMP (*Z*-score = 2.39), CR (*Z*-score = 2.18) and NEW (*Z*-score = 1.82). BGP showed no change in PROG (*Z*-score = 0.05), NEW (*Z*-score = 0.08), NC (*Z*-score = 0.04) and FLARE (*Z*-score = –0.18). BGP showed slight but significant elevation in CR (*Z*-score = 1.30) and IMP (*Z*-score = 1.25). In FLARE, only BAl-p showed elevation (not significant), PICP and BGP stayed at low levels.

Table 2 shows the sensitivity and specificity of each formation marker. The results are similar to those of the *Z*-score analysis.

**Table 1** Values of bone formation markers

| | Without bone metastasis | | With bone metastasis | |
|---|---|---|---|---|
| | **Mean** | **s.d.** | **Mean** | **s.d.** |
| PICP (ng $ml^{-1}$) | 105.8 | 32.03 | 179.4 | 260.65 |
| BA1-p (U $l^{-1}$) | 17.8 | 5.29 | 82.9 | 143.63 |
| BGP (ng $ml^{-1}$) | 4.57 | 3.12 | 5.86 | 6.15 |

A statistically significant difference is shown in PICP and BA1-p between patients with and without bone metastasis.

**Figure 1** The data are expressed as mean ± s.e.m. The *Z*-score in patients with bone metastasis was calculated using the mean and the s.d. of patients without bone metastasis. *Z*-score = (value – the mean of patients without bone metastasis)/s.d. of patients without bone metastasis. CR, complete remission; FLARE, flare-up; IMP, improvement; NC, no change; NEW, new; PROG, progression of disease; PICP, carboxy-terminal propeptide of type I procollagen; BA1-p, bone-specific alkaline phosphatase; and BGP, osteocalcin, so-called bone gla protein. **P* ± 0.05. ***P* ± 0.05 compared with the values in patients without bone metastasis

Figure 2 shows the balance of bone formation and resorption. The difference in Z-scores of BAl-p and ICTP is shown in various conditions (Z-score of BAl-p minus Z-score of ICTP at each point). In NEW, bone formation and resorption was balanced. In PROG, bone formation greatly exceeded bone resorption. In NC, FLARE, CR and IMP, bone formation and resorption were essentially balanced even though bone formation was slightly greater than bone resorption.

**Table 2** Sensitivity and specificity of bone formation markers in bone metastasis

| Reference value | *n* | PICP (170 ng $ml^{-1}$) | BA1-p (30 U $l^{-1}$) | BGP (9.9 ng $ml^{-1}$) |
|---|---|---|---|---|
| Sensitivity (%) | | | | |
| Overall | 96[a] | 31.6 | 52.1 | 19.6 |
| NEW | 9 | 11.1 | 33.3 | 11.1 |
| CR | 15 | 35.7 | 33.3 | 26.7 |
| IMP | 15 | 28.6 | 46.7 | 45.3 |
| FLARE | 3 | 0 | 66.7 | 0 |
| NC | 15 | 31.2 | 60 | 6.7 |
| PROG | 39 | 38.5 | 61.5 | 15.4 |
| Specificity (%) | 46 | 93.3 | 97.8 | 93.5 |

Sensitivity = 100× no. of cases of above reference value with bone metastasis/no. of cases with bone metastasis. Specificity = 100× no. of cases within reference value without bone metastasis/no. of cases without bone metastasis. [a]Several patients were studied repeatedly. The total number of patients is 43. NEW, new; CR, complete remission; IMP, improvement; FLARE, flare-up; NC, no change; PROG, progression.

© Cancer Research Campaign 1997

**Figure 2** The data are expressed as mean ± s.e.m. The values are *Z*-score of BA1-p minus *Z*-score of ICTP. CR, complete remission; IMP, improvement; FLARE, flare-up; NC, no change; NEW, newly diagnosed; PROG, progression of disease; BA1-p, bone-specific alkaline phosphatase; ICTP, pyridinoline cross-linked carboxy-terminal telopeptide. No statistically significant difference is shown

### DISCUSSION

Bone metastasis of prostate cancer is characterized by an excess of osteoblastic activity. Acceleration of bone formation in cases of bone metastasis is no surprise. Various bone metabolic markers have been measured in prostate cancer patients and have been reported (Francini et al, 1988; Shin et al, 1990; Arai et al, 1992; Sano et al, 1994; Koizumi et al, 1995).

The phenotypic developmental sequence of osteoblasts has been divided into three consecutive phases: proliferation, extracellular matrix maturation and mineralization (Stein et al, 1990; Risteli and Risteli, 1993; Zhou et al, 1994). In the early or proliferation phase of osteoblast development, the type I collagen, transforming growth factor-β, and fibronectin genes are actively expressed, and PICP is cleaved off procollagen. Therefore, PICP is believed to be a marker of the early or proliferation phase of osteoblast. In the middle or matrix maturation phase, the expression of alkaline phosphatase mRNA and its protein are increased followed by a rapid decline when the osteoblast enters the mineralization phase. Al-p and BAl-p are considered to be markers of the matrix maturation phase. In the late phase of osteoblast development, osteocalcin (BGP) gene is expressed (Stein et al, 1990; Zhou et al, 1994). Therefore, BGP is considered to be a marker of late bone formation even though the biological role of BGP is not fully understood (Stein et al, 1990; Risteli and Risteli, 1993; Zhou et al, 1994; Calvo et al, 1996).

In the present study, we showed that PICP and BAl-p increased significantly with the progression of bone metastasis. Although BGP increased only slightly with the progression of bone metastasis, the increase was significant in patients with improvement of bone metastasis (IMP and CR). This may suggest that bone formation in the progression of bone metastasis is different from normal bone formation. There is a possibility that BGP may be related to the regulation of bone formation, i.e. BGP gene or protein may be associated with stopping the excess bone formation. In an animal study, Price et al (1982) reported that rats chronically treated by warfarin showed excessive bone formation with growth plate and they suggested the possibility of relationship between the decrease of BGP and excessive bone formation; and recently Ducy et al (1996) reported that osteocalcin gene knock-out mice showed an increase of bone formation without any change of bone resorption or mineralization, resulting in an increase of cortical bone thickness and density – the BGP may be a negative regulator of bone formation. In the progression of bone metastasis, the lack of BGP elevation may be associated with the increase of excess bone formation. With improvement (IMP and CR) of bone metastasis, the elevation of BGP may reduce the excess bone. It is also suggested that the discrepancy in bone formation markers in bone metastasis from prostate cancer may be caused by additional factors, such as cytokines that are secreted from malignant tumours. Further studies, including the search for other related factors, are necessary to clarify the process that underlies bone formation in cases of bone metastasis from prostate cancer.

In practical terms, BAl-p seemed to be the most sensitive formation marker for evaluating bone metastasis from prostate cancer. PICP was not as sensitive as BAl-p in the diagnosis and follow-up of bone metastasis from prostate cancer, and BGP seemed to be difficult to use for the diagnosis of bone metastasis. However, the elevation of BGP may reflect the improvement of bone metastasis.

Histologically, there is much less bone destruction than new bone formation, and there are few osteoclasts but many active osteoblasts surrounded by proliferated stromal cells in the bone metastasis of patients with prostate cancer (Aoki et al, 1986). However, both bone resorption and formation markers increased in patients with bone metastasis from prostate cancer (Koizumi et al, 1995; Kylmala et al, 1995). We also investigated the balance of bone formation and resorption by comparing the *Z*-scores of BAl-p and ICTP. The increase of BAl-p exceeded that of ICTP. However, bone resorption markers seemed to have better correlation with the extent of the bone metastatic burden (Maeda et al, 1997) and a resorption marker was reported to be of prognostic value (Kylmala et al, 1995). Both formation and resorption markers should be measured in the follow-up of bone metastasis from prostate cancer.

In conclusion, we showed that bone formation markers in bone metastasis from prostatic cancer became dissociated. Although the bone formation markers that appeared in early or proliferation phase (PICP) and a marker of middle or matrix maturation phase (BAl-p) were increased in the patients with progression of bone metastasis, a bone marker that appeared in the late bone formation phase (BGP) was not increased with the progression of bone metastasis. BGP was, however, increased in the patients with improvement and complete remission of bone metastasis. Thus, bone formation associated with the progression of bone metastasis from prostate cancer may be different from normal bone formation.

### REFERENCES

Aoki J, Yamamoto I, Hino M, Shigeno C, Kitamura N, Itoh H, Torizuka K, Itoh T and Furuta M (1986) Sclerotic bone metastasis: radiologic–pathologic correlation. *Radiology* **159**: 127–132

Arai Y, Takeuchi H, Oishi K and Yoshida O (1992) Osteocalcin: is it a useful marker of bone metastasis and response to treatment in advanced prostate cancer? *Prostate* **20**: 169–177

Calvo MS, Eyre DR and Gundberg CM (1996) Molecular basis and clinical application of biological markers of bone turnover. *Endocrine Rev* **17**: 333–368

Ducy P, Desbois C, Boyce B, Pinero G, Story B, Dunstan C, Smith E, Bonadio J, Goldstein S, Gundberg C, Bradley A and Karsenty G (1996) Increased bone formation in osteocalcin-deficient mice. *Nature* **382**: 448–452

Francini G, Bigazzi S, Leone V and Gennaric C (1988) Serum osteocalcin concentration in patients with prostatic cancer. *Am J Clin Oncol* **11** (suppl. 2): S3–S87

© Cancer Research Campaign 1997

Francini G, Petrioli R, Manganelli A, Cintorino M, Marsili S, Aquino A and Mondillo S (1993) Weekly chemotherapy in advanced prostatic cancer. *Br J Cancer* **67**: 1430–1436

Koizumi M, Yamada Y, Takiguchi T, Nomura E, Furukawa M, Kitahara T, Maeda H, Takahashi S, Aiba K and Ogata E (1995) Bone metabolic markers in bone metastasis. *J Cancer Res Clin Oncol* **121**: 542–548

Kylmala T, Tammela TLJ, Risteli L, Risteli J, Kontturi M and Elomaa I (1995) Type-I collagen degradation product (ICTP) gives information about the nature of bone metastasis and has prognostic value in prostate cancer. *Br J Cancer* **71**: 1061–1064

Maeda H, Koizumi M, Yoshimura K, Yamauchi T, Kawai C and Ogata E (1997) Correlation of bone metabolic markers and bone scan in prostate cancer. *J Urol* **157**: 539–543

Pollen JJ, Witztun KF, Ashburn WL (1984) The flare phenomenon on radionuclide bone scan in metastatic prostate cancer. *AJR* **142**: 773–776

Price PA, Williamson MK, Haba T, Dell RB and Lee WS (1982) Excessive mineralization with growth plate closure in rats on chronic warfarin treatment. *Proc Natl Acad Sci USA* **79**: 7734–7738

Risteli L and Risteli J (1993) Biochemical markers of bone metabolism. *Ann Med* **25**: 385–393

Sano M, Kushida K, Takahashi M, Ohishi T, Kawana K, Okada M and Inoue T (1994) Urinary pyridinoline and deoxypyridinoline in prostate cancer patients with bone metastasis. *Br J Cancer* **70**: 701–703

Shin WJ, Wiertzbinski B, Collins J, Magoun S, Chen IW and Ryo UY (1990) Serum osteocalcin measurements in prostate carcinoma patients with skeletal deposits shown by bone scintigram: comparison with serum PSA/PAP measurements. *J Nucl Med* **31**: 1486–1489

Slack MH and Murrhy GP (1984) Criteria for evaluating patient response to treatment modalities for prostatic cancer. *Urol Clin N Am* **11**: 337–342

Stein GS, Lian JB and Owen TA (1990) Relationship of cell growth to the regulation of tissue-specific gene expression, during osteoblast differentiation. *FASEB J* **4**: 3111–3123

Zhou H, Choong P, McCarthy R, Chou ST, Martin TJ and Ng KW (1994) In situ hybridization to show sequential expression of osteoblast gene markers during bone formation in vivo. *J Bone Miner Res* **9**: 1489–1499

 *© Cancer Research Campaign 1997*